# Qualitative Assessment of Risk for Monkeypox Associated with Domestic Trade in Certain Animal Species, United States

**DOI:** 10.3201/eid1212.060454

**Published:** 2006-12

**Authors:** Susan M. Bernard, Steven A. Anderson

**Affiliations:** * US Food and Drug Administration, College Park, Maryland, USA

**Keywords:** monkeypox, risk assessment, communicable diseases, perspective

## Abstract

In 2003, US officials identified several human monkeypox cases and traced the virus exposure to infected captive prairie dogs. The virus was likely introduced through a shipment of imported African rodents, which were kept with other mammals, including prairie dogs, in a pet distribution facility in the Midwest. To prevent the further introduction and spread of the virus, federal agencies restricted the importation of African rodents and restricted the domestic trade or movement of prairie dogs and certain other rodents. In this qualitative assessment of the risk for monkeypox associated with the 2003 outbreak, we conclude that the probability of further human infection is low; the risk is further mitigated by rodent import restrictions. Were this zoonotic disease to become established domestically, the public health effects could be substantial.

## Abstract 2

TOC summary: The probability of further human infection is low and the risk is further mitigated by rodent import restrictions**.**

In May and June 2003, public health officials identified an outbreak of human monkeypox in the United States (*1**–**3*). This was the first instance of human monkeypox virus (MPXV) infection detected outside its endemic range in Africa (*3*). As of July 30, 2003, a total of 72 human cases had been reported (*4**,**5*). Thirty-seven (51%) cases were eventually laboratory confirmed, and 35 met the case definition set by the Centers for Disease Control and Prevention (CDC) (*4**,**5*). Among the 35 patients whose cases were laboratory confirmed before July 11, 2003 (*1*), 32 (91%) tested positive for MPXV by PCR, culture, immunohistochemical testing, or electron microscopy of skin lesions; 2 tested positive by PCR and/or culture of an oropharyngeal or nasopharyngeal swab; and 1 tested positive by PCR and culture of a lymph node aspirate (*1*). To date, no new animal or human cases have been reported.

The outbreak was relatively large compared with most reported events in Africa, but clinical features were milder than typically seen there (*3**,**6**,**7*). No human deaths occurred (*1**,**8*), although 2 children required intensive care (*1**,**8*). One patient received a corneal transplant due to chronic ocular infection (*8*).

Most patients were exposed to prairie dogs, primarily from an Illinois animal distributor (IL-1). Most of those infected had direct physical contact with infected animals; infection likely resulted from bites or scratches or through open wounds (*1**–**3*). Some patients were exposed to premises where prairie dogs were kept (*1*).

Traceback implicated rodents from a shipment of African animals imported to Texas on April 9, 2003, as the probable source of MPXV (*1**,**4*). The shipment contained ≈800 small mammals of 9 different species, including 6 genera of African rodents (762 rodents total): rope squirrels, tree squirrels, Gambian giant rats, brushtail porcupines, dormice, and striped mice (*1**,**2*), as well as cusimanses, genets, and palm civets (*9*). Rodents from the shipment were housed with or in close proximity to prairie dogs at IL-1. Approximately 200 prairie dogs were at IL-1 coincident with the arrival of the imported African rodents (*1*). Many prairie dogs from IL-1 were distributed to other states for sale as pets (*1**,**4*). CDC traced 93 infected or potentially infected prairie dogs from IL-1 (*1*). An additional, unknown number of prairie dogs died or were sold at animal swap meets for which records are not available (*1*) (Table 1).

**Table 1:** Disposition, as of July 11, 2003, of African rodents imported from Ghana to the United States on April 9, 2003*

Rodents	Dead†	Alive	Lost to follow-up	Total (n = 762)
Gambian giant rats	26	20	4	50
Dormice	≈350	27	≈135	≈510
Rope squirrels	49	4	–	53
Tree squirrels	24	20	3	47
Striped mice	14	50	36	100
Porcupines	2	–	–	2

*Source (*1*). †Includes animals that died of monkeypox and those that have been euthanized.

To prevent the introduction and spread of infected animals into susceptible populations, on June 11, 2003, the Food and Drug Administration (FDA) and CDC issued an order that prohibited 1) importation of all rodents from Africa and 2) transportation, sale, or any other commercial or public distribution, including release into the environment, of prairie dogs or rodents from 6 species represented in the African shipment (*10*). On November 4, 2003, FDA and CDC published an interim final rule (*11*) that imposed import restrictions on all African rodents and established or modified restrictions on the import, capture, transport, sale, barter, exchange, distribution, and release of prairie dogs, the 6 imported species, and possibly, by order, other animals with the potential to transmit MPXV. Neither CDC nor FDA exercised its statutory authority to seize and destroy animals to prevent the spread of MPXV.

We prepared this qualitative risk analysis to help understand the impact of the domestic trade restrictions on the current risk for human monkeypox infections. We evaluated the data and uncertainties concerning monkeypox and its potential spread to animal and human populations and characterized the probability of harm on the basis of those data. Because of CDC's import restrictions on all African rodents, we did not estimate the risk posed by importation of animals into the United States. We focused only on monkeypox and did not consider other zoonotic agents that might be transmitted by the species discussed. This risk assessment follows a generally accepted 4-part framework (*12*). The hazards are MPXV and its potential for transmission and spread from animals to humans; the risk is human infection from prairie dogs and possibly imported rodents.

## Hazard Identification

Human monkeypox is a sporadic zoonotic viral disease, caused by an orthopoxvirus that until 2003 was known to have occurred only in parts of Africa (*3**,**7**,**13*). The first human illness was identified in 1970 in a child (*7**,**14*). Previous cases were likely mistaken for smallpox (*14*). Although it was first isolated from a captive primate (*3**,**6*), rodents are its likely primary natural reservoir (*7**,**15**–**17*); its complete mammalian host range is unknown. The mode of transmission between infected animals and humans is not well defined (*18*). Direct mucocutaneous contact and respiratory routes have been implicated in epidemiologic and experimental research (*15**,**18**,**19*).

The estimated mean human incubation period is 12 days (*1**,**3**,**15**,**16*). The disease is characterized by a rash similar to that observed with smallpox (*14*) or chickenpox (*10**,**15**,**20*). The infectious period occurs during the first week of the rash (*7*); symptoms include headache, fever, sweats, and severe lymphadenopathy (*15**,**16**,**20*). Among African patients with a history of smallpox vaccination, monkeypox is usually milder with lower numbers of deaths (*3**,**6**,**16*). Subclinical or very mild infection can occur in humans (*16**,**21**,**22*).

Case-fatality rates in African outbreaks range from 4% to 33% (*6**,**23*) and are high among children (*3**,**6**,**14**,**23*). Variability in case-fatality rates may reflect incomplete assessment of the total number of cases, variations in case definition, and variability in the virulence of MPXV strains. The US outbreak has been associated with a milder strain (*3**,**24**–**26*). Case fatality also likely depends on differences in exposure, susceptibility, and healthcare (*14*).

Repeated animal reintroduction of MPXV is believed necessary to endemic infections in human populations. Human cases in disease-endemic areas tend to be sporadic and isolated and primarily associated with direct animal-to-human transmission (*24**,**27*). However, clusters associated with common source and human-to-human transmission occur and may in Africa be increasing with decreased prevalence of prior smallpox vaccination (*6**,**7**,**15**,**16**,**28**,**29*).

FDA has not approved a treatment for monkeypox. Suggested treatment options include cidofovir (*30**–**32*). Efficacy of vaccinia immune globulin in humans has not been established (*30**,**31*). After the onset of symptoms, supportive therapy is usually the recommended treatment (*31*). Preexposure and postexposure smallpox vaccine was used during the 2003 outbreak, with only relatively minor adverse events reported (*1**,**32*).

## Hazard Characterization

Much is unknown about pathogenesis and transmission dynamics of MPXV in humans and animals. Limited research suggests that at least in some host mammals latent or inapparent infection occurs (*15*). In addition to serologic evidence of orthopoxvirus exposure, MPXV has been recovered from the kidneys of healthy-appearing animals (*15**,**16*). The latency period is unknown, as is whether the virus can be transmitted during such periods.

The complete host range of MPXV in Africa is unknown. Animal antibody surveys in disease-endemic areas suggest infection is enzootic among squirrels, other rodents, and monkeys, although other animals may be infected (*6**,**15**,**16**,**33**–**35*).

The number of animals exposed or infected in the United States is unknown and impossible to estimate. Approximately 800 animals were recorded in the African shipment, but disposition information is available only for rodents (Table 1) (*1*). A Gambian giant pouched rat, 3 dormice, and 2 rope squirrels from the shipment were tested and found to be infected with MPXV (*1*). Infected animals from the shipment were housed or transported with prairie dogs and other mammals. An unknown number of prairie dogs and animals from other species became infected. Although many prairie dogs became ill and several died, some infected animals survived. The secondary attack rate among susceptible animals is unknown and cannot be estimated with available data.

CDC necropsied 249 animals involved with the outbreak, confirming infection in 33 animals with PCR (*36*) and in 22 animals through virus isolation from various tissues. Infection was confirmed in 14 prairie dogs, 2 Gambian giant pouched rats, 9 dormice, 3 rope squirrels, 1 ground hog, 1 hedgehog, 1 jerboa, and 2 opossums.

CDC performed extensive histopathologic examination on 2 necropsied prairie dogs from IL-1 and detected MPXV DNA by using real-time PCR (*18*). The necropsied prairie dogs had MPXV in saliva, lesion exudates, and bronchi and lung parenchyma (*18*). Approximately 110 of the ≈200 prairie dogs likely exposed at IL-1 were sold after the African animals were introduced and before 15 of the prairie dogs at IL-1 became ill. Ten of the ill prairie dogs died rapidly (*1**,**3**,**18*).

In June 2003, CDC evaluated an unspecified number of prairie dogs, dormice, hedgehogs, jerboas, opossums, and numerous other species (a total of 18 species) from IL-1; of these, 2 prairie dogs, 7 dormice, 1 African hedgehog, 1 jerboa, and 1 gray short-tailed opossum tested positive for MPXV by PCR (*36*). When these animals were infected or if they could transmit disease is not known.

On June 19, 2003, CDC acquired 61 live animals from the original shipment. On August 20, 2003, CDC acquired from the state of Illinois 291 animals remaining at IL-1, including African and domestic species. Numerous other animals were acquired from Iowa, Wisconsin, Indiana, and Ohio. Of 172 animals tested from the various states as well as from the original shipment, 25 showed serologic evidence of infection without overt signs of disease (i.e., PCR and tissue culture negative). On June 24, 2003, an oral and ocular swab from a dormouse from IL-1 tested positive by PCR. After the dormouse died a month later, its tissues tested positive for MPXV by PCR and culture. A second dormouse from IL-1 that also tested positive in June appeared healthy; however, when it was euthanized in December 2003, swabs and necropsy samples of various tissues, urine, and feces were positive by PCR. No viral antigen was detected on pathologic examination of tissues (*36*).

Investigations of human cases from the outbreak support the hypothesis that close direct contact with infected animals was necessary for infection. Cases occurred among persons who were bitten by infected prairie dogs or infected through open wounds (*3**,**8*). The 11 Wisconsin patients included a child and parents; a meat distributor who also distributed exotic animals; his wife; 2 employees of 2 different pet stores; 2 veterinarians from different clinics; a person who had bought prairie dogs; and that person's houseguest. All of these patients reported direct contact with an infected prairie dog (*3*), although human-to-human transmission could not be ruled out for the parents (*3*).

Data on duration of infection are limited. Virus appears to be present in some animals months after infection, regardless of clinical illness. In addition to CDC's data on dormice, data derived from experimental infection of small numbers of laboratory animals documented infectious MPXV in tissues 3–6 weeks after exposure (*18*). Clinical and asymptomatic infections have been reported among captive primates; severity varied depending on the species and route of inoculation (*16*). CDC has reported elevated tissue viral loads in 2 necropsied prairie dogs (*18*). In another study, 10 experimentally infected North American ground squirrels died within 9 days, although no obvious signs of disease except for lethargy and anorexia developed (*37*). Squirrels infected intranasally had a longer incubation period and later death (*36*). Ten prairie dogs infected experimentally with a human MPXV isolate were highly susceptible to infection but had a lower death rate and less severe pathologic change than were seen in the squirrel study that used the same dose (*19*).

A human adult infected during the 2003 outbreak experienced keratitis and corneal ulceration as a complication of infection and ultimately received a corneal transplant (*8*). Corneal ulceration has also been reported in some African patients (*16*).

## Exposure Assessment

In African outbreaks, capturing, handling, and eating wild animals have been associated with infection (*6**,**23**,**34*). In the United States, monkeypox occurred in humans who had direct contact with infected animals and were bitten or infected through open wounds (*3*). These persons included pet dealers, pet owners and their children, and contacts of these people at risk of coming into direct contact with the infected animal. Although potential exposure occurred in settings that included pet stores, swap meets, and wild animal trade centers (*1*), no evidence exists that persons casually exposed to infected animals were infected. The magnitude and scope of this pet trade are not well quantified. In 2002, ≈30,000 prairie dogs were sold at pet dealers, swap meets, flea markets, and other venues open to the public (*11*).

Of the 762 rodents in the African shipment, CDC traced 584 (77%) (*1*). The remaining 178 (≈23%) could not be traced beyond the point of entry (*1*). The fate of the 50 nonrodent animals on the shipment is unknown. Of the ≈200 prairie dogs that may have been exposed to MPXV at IL-1, 107 (54%) have not been accounted for. These animals will not likely be traced. A small number of animals associated with the outbreak, including some known to have been infected, are in the possession of pet dealers and private owners; their capacity to transmit infection is unknown. Animals from species other than the listed species—gerbil, hamster, chinchilla, opossum, groundhog, hedgehog, and jerboa—were discovered to be infected, although no confirmed human cases of infection were associated with contact with any animal except prairie dogs (*1**,**9*).

To evaluate the potential spread of the disease beyond the initially exposed animals, the US Geological Survey's National Wildlife Health Center trapped 237 small mammals from 14 species at 9 sites in Wisconsin and Illinois where cases of monkeypox were reported. All were negative for monkeypoxvirus or monkeypoxvirus-specific antibodies (*38*). These small amounts of data are insufficient to establish the absence of MPXV in the wild.

The federal restrictions on importation of high-risk species and trade in the listed species have likely substantially reduced the potential risk for exposure of uninfected animals or persons to MPXV. However, some residual risk for MPXV infection through illegal importation or infection in legally imported, nonlisted species may exist.

## Risk Characterization

Table 2 provides summary information on the qualitative variables considered in the risk characterization. We evaluated the probability of human monkeypox infection that resulted from certain types of exposure or contact (direct or indirect) to animals (infected or noninfected) and qualitatively estimated the probability and, to a lesser extent, the possible severity of infection. Most confirmed human cases in the United States were associated with direct, close contact with infected prairie dogs. We characterize as type I direct contact with the animal and as type II direct contact that also involves bites, scratches, or other contact with the mucous membranes or nonintact skin of the affected person. Infection through aerosolized particles without direct animal contact, or by some other less direct method, as well as human-to-human transmission, cannot be ruled out. The probability of infection is dependent on whether the animal is infectious (shedding virus) and varies with the level of shedding and the nature of human-animal interactions (type, frequency, and duration of contact). We assume, on the basis of the data described above, that the primary means of transmission affecting the risk would be from animal to human.

**Table 2:** Variables considered in characterizing risk for human monkeypox cases and the degree of uncertainty associated with these variables

Variable	Degree of uncertainty
Animal host and carrier species	High—some, but not all, host species identified
Proportion of probable host or carrier species infected with virus	High—need to assume absent data that all animals within known or probable carrier species are infected
Proportion of animals exposed during US 2003 outbreak infected with virus	High—need to assume that all exposed animals are infected
Susceptibility of naive animals to infection	High—but experience in United States and Africa suggests several species and orders can be infected with monkeypox virus
Latency in nonhuman species	High
Duration of infection or infectiousness in nonhuman species	High
Seasonality of disease	High—some indication of peak monkeypox cases in humans in July and August in African outbreaks, which may be associated with human behavior rather than characteristics of virus or host animals
Incubation in nonhuman species	High
Infection rates in exposed nonhuman species	High
Proportion of infected animals (of different species) that shed virus	High
Mode(s) of transmission across species and to humans	High—but evidence of mucocutaneous and respiratory transmission pathways
Attack rates among humans exposed to infected animals	High
Secondary attack rates among humans	High—secondary attack rates seem to be increasing in monkeypox-endemic areas due to increasing susceptibility of exposed populations, and historical data indicating low risk for human transmission may be unreliable
Fatality rates in nonhuman species	High

Several categories were established to define and qualitatively characterize the risks. Low risk denoted no direct human contact with a captive animal(s); even if animal infection status was unknown or the animal was infected, the exposure to the animal was likely insufficient for animal-to-human disease transmission to occur. Medium risk described human contact that was direct but the exposure involved type I contact with a potentially infected animal or animal(s) of unknown infectious status. High risk designated direct human contact and involved a type II contact with a captive animal(s) infected with MPXV or with unknown infection status but likely MPXV exposure. Finally, the term severity of infection and illness denoted any individual infection with MPXV that should be considered serious and potentially fatal. The risk to the persons and the risk for the spread of the disease to others made MPXV infection a potentially serious public health matter.

The probability that any surviving animal directly involved in the outbreak may be infected must be considered high, given the possibility of latent infection. Making the unlikely assumption that all of these animals are still alive, the group includes the 178 African rodents, mostly dormice, lost to follow-up, 107 prairie dogs from IL-1 that were not traceable, and 50 nonrodent animals included in the African shipment. An unknown number, but clearly most, of the affected African rodents and IL-1 prairie dogs that were traced and identified as alive as of July 2003 (121 African rodents and ≈93 prairie dogs) have since died or been euthanized.

Some animals from other species that were in the affected pet distribution facilities during the outbreak tested positive for MPXV. An unknown number of these exposed animals are likely to be alive and in private or commercial ownership; what proportion of these animals is infected with MPXV is unknown but is assumed to be small. No confirmed cases of human infection or further cases of animal infection have been associated with these animals. However, all animals directly associated with the 2003 outbreak should be considered to pose a continued high risk for infection.

The probability of infection in rodents or other animals imported from monkeypox-endemic regions is unknown. Imported African rodents were almost certainly the source of the US outbreak. Animals imported as pets are handled by several persons as they pass from importer to owner, and they may be housed and transported in close proximity with nonimported susceptible animals. Current import restrictions on African rodents substantially reduce the risk for introduction and spread of MPXV, but a potential residual risk remains because of illegal importation as well as import of nonrestricted species that may carry the virus. Some previously imported animals from restricted species might also be infected with MPXV, although this risk is unknown and assumed to be extremely low.

For domestically bred African rodents, the risk they may pose of transmitting MPXV to humans depends on the risk that the rodents will be exposed to infected animals. Absent a tracking or pedigree system that distinguishes domestically bred from imported, wild-caught animals is impossible. Trade in domestically bred African rodents could increase the risk for human infection if illegally imported infected animals are identified as captive-bred. The monkeypox risk to humans posed by prairie dogs is a function of the animals' possible contact with infected animals and their potential for viral transmission.

The number of animals infected with or exposed to MPXV in the outbreak that might still be alive is likely small. However, these animals may be widely distributed geographically, and they may have spread the virus to other animals not currently known to pose a risk. The risk for MPXV infection and spread among prairie dogs are mitigated by current import and trade restrictions and the death or euthanization of most animals directly associated with the outbreak. The probability that an uninfected prairie dog will come into contact with an infected captive or released animal and that there will be sufficient exposure for infection is likely low. If such contact occurs, however, these animals are highly susceptible.

Little evidence about the MPXV status of wild prairie dogs exists. Given the high rates of illness and death among captive prairie dogs exposed to MPXV in 2003, anticipating that the virus would result in a die-off that would be detected may be reasonable; however, in addition to the lack of data, uncertainties about the virus and the susceptibility of the animals in the wild preclude drawing any conclusions.

The risk for new domestically acquired human cases is low with the current restrictions on import and trade in certain species in place. No new cases have been reported in humans or animals since the outbreak, despite the likelihood that some surviving infected animals have been kept alive by individual or commercial owners. Limited surveillance efforts have not identified MPXV in wild animal populations; however, the virus could possibly become enzootic here if an infected animal were released or escaped into the wild and spread the virus to susceptible mammals. Were that to occur, human cases would likely result. The risk that monkeypox could become enzootic is relevant in evaluating the risk of importing potential mammalian carriers of MPXV or in allowing contact between likely carriers and susceptible domestic mammals.

Data limitations preclude quantitative, and limit accurate qualitative, estimation of the human risk for monkeypox in the United States (Table 3). Research is needed on disease dynamics, range of host species, and the parameters of wild animal trade and ownership.

**Table 3:** Qualitative estimation of risk to humans*

Human exposure to animal	Animal infected	Infection status of animal unknown	
		Exposure likely	Exposure unlikely
Indirect exposure/no direct contact	Low	Low	Low
Direct contact, type I* (direct contact without type II exposure)	Medium	Medium	Low
Direct contact, type II* (bite, scratch, or contact of animal’s body fluid with mucous membrane or nonintact skin)	High	High	Low

*Risk was based upon type of exposure to an animal and the infection status of the animal. Type I and type II are arbitrary classifications.
